# The State of Lunacy in Great Britain

**Published:** 1862-10

**Authors:** 


### Art. VI. — THE STATE OF LUNACY IN GREAT BRITAIN.

### I.—England.

The asylum population of England, according to the 16th, and latest, Report of the Commissioners in Lunacy, amounted, on the 1st January, 1862, to 26,200. This is an increase of 1355 on the number of lunatics remaining in the different classes of asylums on the 1st January, 1861. The augmentation of private patients within the twelve months of 1861 was 128; of pauper, 1177.

In the four years 1858-61 the increase of asylum population had been as follows:—

| | Private. | Pauper. | Total Increase. |
|---|---|---|---|
| 1858 | 296 | 602 | 898 |
| 1859 | 96 | 768 | 864 |
| 1860 | 189 | 924 | 1113 |
| 1861 | 128 | 1227 | 1355 |

The total number of pauper patients on the 1st January, 1862, was 20,950, of whom 1562 were in private asylums and hospitals.

The existing and prospective accommodation for patients, in County and Borough Asylums, on the 1st January, 1858, was 21,048. If we estimate the additions, completed or in progress, since this calculation was made, at 2000, the present accommodation would be a little above 23,000. The annual average rate of increase of pauper lunatics in County and Borough Asylums was, in the five years 1857—61, 1087. At this rate of increase, the existing amount of accommodation in these asylums would be exhausted by pauper patients alone in about three years.

The pauper patients in Hospitals and Licensed Houses amounted, on the 1st of January, 1862, to 1562.

The increase of lunatic population in County and Borough Asylums, during 1861, was 1062 (private patients, 55; pauper, 1008); in Hospitals, 2 (the private patients having decreased 2, the pauper increased 4); in Metropolitan Licensed Houses, 179 (private patients, 57; pauper, 122); in Provincial Licensed Houses, 111 (private patients, 18; pauper, 93).

The chief bulk of the Commissioners' Report for 1861 is occupied with an account of the history of the past and present condition of the Provincial Licensed Houses. In their fourteenth Report the Commissioners gave a similar history of the Metropolitan Licensed Houses. An appendix to the present Report contains copies of the entries made in the visitors' books of the different County and Borough Asylums by the Commissioners in Lunacy at their latest visits in 1861.

The statements of the Commissioners show that the condition of the great majority of both licensed houses and county and borough asylums is most excellent. Of one licensed house only is it said that the arrangements and management are thoroughly unsatisfactory; and the number of those houses of which the Commissioners express themselves somewhat doubtfully is very few.

Respecting the Public Asylums, the Commissioners state, that their visits and recommendations "have led to important improvements;" and that "the beneficial effects upon the patients have been obvious, and recognised by the authorities immediately responsible for their care and treatment."

The enormous establishments of Hanwell and Colney Hatch have again obtained unenviable notoriety from the occurrence of a death from the brutality of an attendant, and the probable death of a second patient from a similar cause in the former, and the murder of one patient by another in the latter. On these cases the Commissioners remark, "The experience of such occurrences, indeed, furnishes the decisive argument against lunatic establishments of the magnitude of Colney Hatch and Hanwell. Where persons requiring constant protection are brought together in such great numbers, it is impossible to obtain for the individual the protection required, from the absence of that continual watchfulness in the particular case which is indispensable to secure it." The Commissioners express the opinion that the Secretary of State should be entrusted with increased powers over the management of county asylums.

Plans and descriptions are appended to the Report of the asylums which have recently been erected for the City of Bristol, the County and Borough of Cambridge, the joint counties of Cumberland and Westmoreland, and those of Beds, Herts, and Hants. The details given are too extended to admit of quotation; but a few of the more important facts may be referred to.

The City of Bristol Asylum has attached to it about seventeen acres of ground, the whole of which is used as a vegetable garden. The building is designed to receive 200 patients; and accommodation is provided for one-third of the number in single rooms, the remainder being placed in associated dormitories, which will each hold from six to eleven inmates. A few of the single rooms are padded. The infirmaries are arranged to hold 22 patients. The whole building is constructed as nearly fireproof as possible. The large dormitories are calculated to give rather more than 500 cubic feet for each patient; in the infirmaries the space allotted to each patient is somewhat more. All the corridors for patients are 12 feet wide, provided with open fireplaces, and form exceedingly cheerful galleries commanding good views of the picturesque country around. In the day-rooms upwards of 300 cubic feet has been allowed for each patient, and the large rooms have two fireplaces.

The minimum cubic space recommended by the Barrack Commission for each man in barrack-rooms is 600 cubic feet; in hospital 1200. Adopting these data, the cubic space allowed for patients in the Bristol Asylum would appear to be somewhat small. The cost of the Asylum was £27,500; style, Italian; structure, stone.

The County and Borough of Cambridge Asylum is built for 252 patients, 126 of each sex. The accommodation consists of 26 single rooms, and 50 beds in dormitories on the ground floor; 26 single rooms, and 98 beds in dormitories on the first floor; and 10 single rooms, and 42 beds in dormitories on the second floor. There are about 61 acres of land attached to the building, and laid out as garden and farm. The cost of the building is not stated; the style is a modification of the architecture of James's period; and the construction is of white brick, relieved by red and black brick, and with stone dressings.

The Cumberland and Westmoreland Asylum is built upon a property containing 108 acres, and which cost about £8000. The building is adapted for 200 patients, and is described as nineteenth-century Italian in style. The following extracts from the architect's description will show the nature of the accommodation for the patients, excepting the dining and recreation hall (60 feet by 30 feet) :—

"The corridors or exercise galleries are each 83 feet 6 inches long and 10 feet wide, at the ends of which, and separated by a glazed screen, are the day-rooms. The corridors are lighted by a series of windows with arched heads, giving an agreeable arcaded appearance, and the glazed screens at the ends, separating the day-rooms, have been adopted in order to remove the depressing effect found to be produced on the inmates by a dead wall, which gives an impression of restraint and confinement.

"The day-rooms are 30 feet by 20 feet, well lighted by windows, looking out in three different directions.

"Each gallery and each day-room has two open fireplaces.

"The position of the attendant's room at the end of the gallery affords ample means of inspection through the whole of the ward, and every person entering the ward, or passing from it, either to the covered ways, dining-hall, or airing-courts, must come under his observation.

"Ten separate sleeping-rooms, each 9 feet by 7 feet, lighted from the north side, are entered from the corridor, as indicated on the plan.

"At the angles adjoining the day-rooms are the principal stone staircases, 5 feet wide; the wells being built up to form ventilating shafts, up the centre of which are the copper flues from heating apparatus in the basement, more particularly described hereafter.

"A projecting building to the north, behind the staircase, contains the bath-rooms, lavatories, slop-rooms, water-closets, &c., and which are all grouped together, in order that all the piping may be at once led to the outlet drain.

"One great advantage contemplated in this arrangement has been the complete separation of these offices from the wards, and the means by cross ventilation of cutting off any effluvia from the main building.

"The infirmary is situated in a small wing, projecting southwards at right angles with the corridor and general day-rooms, and contains day-room, 18 feet by 13 feet; attendant's room 13 feet by 10 feet; store-room, lavatory, and water-closet, with three separate sleeping-rooms 10 feet by 7 feet, having a short corridor and independent access to the airing-court.

"The arrangement of the whole of these wards on the two sides appropriated to males and females is exactly the same, and the floor above is in all respects similar.

"The plan varies, however, on the top story, which is entirely devoted to associated dormitories for 54 inmates. Each ward on this floor has four large sleeping apartments, the attendants' rooms being so placed as to give the means of inspection. The bath and water-closet arrangement is again repeated on this floor, as on the two floors below. . . . .

"Each sex has one large airing-court on the south side of the building, the entire length of the ward occupied by the patients extending about 20 yards beyond the angle of the building, and returning as far as the back of the wards respectively, as indicated on the plan. The division between the male and female airing-court is formed by a garden in front of the dining-hall. The fence walls are sunk in a deep trench or cutting, so that the wall has only the appearance of a low parapet above the level of the court and walks.

"Small separate courts are also provided for the working patients adjoining the workshops and laundries.

"The convalescent or working patients are entirely separated from the main building in detached pavilions or blocks on the northern side.

"The block occupied by the males has a day-room and dormitory for 4, with attendant's room on the ground floor, and a dormitory for 12, with attendant's room on the first floor; also a large timber-yard, shops for cabinet-makers, joiners, plumbers, painters, shoemakers and tailors, a bakehouse, flour-store, water-closets, lavatories, slop-room, &c. The stable, coach-house, and a *post mortem* or dead-house, adjoin, with a separate stable-yard.

"The block occupied by the females contains the wash-house and laundry, each 40 feet by 20 feet; a drying closet, rooms for the reception and distribution of the clothes, each 20 feet by 10 feet, with boiler-house adjoining; also a day-room, attendant's room, a dormitory for 4 on the ground floor, and dormitory for 12 and attendant's room on the floor above.

"The wash-house and laundry are lofty and well lighted, and adjoining is a drying-yard in addition to the laundry court. . . . .

"Finding that the results of the many costly artificial systems of ventilation resorted to in large asylums and gaols had been practically very uncertain in their success, the architect recommended to the Committee of Visitors the adoption of open fires, and windows opening at the top and bottom, as the most natural means of attaining a healthful and agreeable warmth with the opportunity of easy and rapid ventilation.

"Thus, when the patients go out into the airing-courts, by opening the windows in the exercise galleries and those in the separate sleeping-rooms, a thorough change of the air in each ward is effected in a very short time.

"Over the door of each separate sleeping-room is hung a small pivoted window in order to assist the ventilation of these rooms during the night. In the roof, over each ward, is a horizontal ventilating trunk for carrying off the vitiated air, to which are connected a number of small flues from the several apartments.

"These horizontal flues discharge into the upright ventilating shafts before referred to, in the centre of the great staircases, and the foul air thus passes off from the building. These upright ventilating shafts or towers rise about forty feet above the ridge of the roof of the main building, and are of sufficient size to contain a circular copper tube or smoke-flue from the furnace of the bath-boiler in the cellar. This smoke-flue thus becomes a heated core up the centre of the air shaft, and by rarefying the air about it creates a continual rising current, and extracts the air from the horizontal shafts in the roof and from the several air-flues connected therewith.

"The ventilation of the large dormitories on the top floor has been assisted by inserting a number of air gratings communicating with the outside of the level of the floor."

The asylum is built from a design and plans prepared by Mr. Thomas Worthington, of Manchester, carried out under the direction of Mr. Cary, the county architect and surveyor. The building and fixtures have cost about £25,000, exclusive of gas-works, fencing, bridges, lodge, and other matters external to the building, which have cost about £6000.

The Joint Counties Asylum of Beds, Herts, and Hants stands upon an estate of upwards of 250 acres. "The arrangements for the wards are exceedingly simple; they are short, with corridors twelve feet wide, in which are single bed-rooms for patients, and attendants'-rooms, lavatory, &c. No. 1. corridor is headed by a congeries of three day and dining rooms, communicating with each other, and yet can be entered separately. These, with the corridor, form a recreation hall. The upper floors consist entirely of sleeping-rooms." The building is constructed of red and white brick, with stone quoins in parts. The amount of accommodation is not stated. Plans are prepared for erecting double cottages on the estate for thirty or forty patients, if deemed expedient.

. . . .

The attention of the Commissioners has been directed to the unsatisfactory condition of the insane in the Channel Islands and the Isle of Man, over which they have no jurisdiction. The representations made by them to the Secretary of State will no doubt lead to an amelioration in the condition of lunatics in both localities.

Several Acts were passed in the course of the Parliamentary Session of 1861 affecting lunatics.

By the "Act for the Abolition of Contributions by Counties for the Relief of Prisoners in the Queen's Prison, and for the Benefit of Bethlehem Hospital" (24 Vict. c. 12), the Secretary of State is no longer empowered to order the removal of insane prisoners from the Queen's Prison to Bethlehem Hospital.

The "Act to amend the Laws regarding the Removal of the Poor and the Contribution of Parishes to the Common Fund of Unions" (24 & 25 Vict., c. 55, s. 6, 9), provides for the maintenance of pauper lunatics in an asylum out of the common fund of the union to which their parish may belong, and for a more equitable arrangement of the contributions of parishes to the common fund of the union. This will prove a great relief to poor parishes, and remove one of the incentives for keeping pauper lunatics out of asylums.

The "Act to amend the Law relating to Bankruptcy" (24 & 25 Vict., c. 134, s. 109), empowers justices of peace to remove lunatic debtors from gaols to asylums.

The tables on the next page show more fully the state of the asylum population in 1861.

*Criminal Lunatics.*—The Judicial Statistics for 1861 contain many interesting details respecting criminal lunatics, for the twelve months ending the 29th September, 1861. At that time the number of criminal lunatics under detention was 799. At the beginning of the year the number had been 776; during the year 135 were committed, and 59 "received from other asylums," making a total of 970, of whom 750 were males, and 220 females. Of this number there were discharged, or removed by death or otherwise, 134 males and 37 females, making a total of 171. The manner of discharge or removal was as follows:—

| | |
|---|---|
| Died . . . . . . . . . | 49 |
| Escaped . . . . . . . . | 4 |
| On becoming sane . . . . . . | 40 |
| Removed, sane, for trial or punishment . . | 20 |
| Removed to other asylums . . . . . | 58 |

An increase of 23 or 2·9 per cent. appears in the number remaining at the close of the year, as compared with the number under detention at its commencement, there having been an increase of 581, or 7·5 per cent., in 1860, following an increase in each of the three preceding years. The increase in the number remaining under detention at the close of last year, as compared with 1856, amount to 202, or 33·8 per cent.

| | Number of Patients, 1st January, 1861. | | | | | | | | | | Discharges during the Year 1861. | | | | | | Deaths during the Year 1861. | | | | | | | | |
|---|---|---|---|---|---|---|---|---|---|---|---|---|---|---|---|---|---|---|---|---|---|---|---|---|---|
| | Private. | | | Pauper. | | | Total Lunatics. | Admissions during the Year 1861. | | | Total Number. | | | Number Recovered. | | | Total Number. | | | From Suicide. Act committed in Asylum. | | | From Suicide. Act committed before admission. | | |
| | M. | F. | Total. | M. | F. | Total. | | M. | F. | Total. | M. | F. | Total. | M. | F. | Total. | M. | F. | Total. | M. | F. | Tot. | M | F. | Tot. |
| County and Borough Asylums . | 108 | 104 | 212 | 8,269 | 10,111 | 18,38c | 18,592 | 3,086 | 3,182 | 6,268 | 1,365 | 1,723 | 3,088 | 958 | 1,262 | 2,220 | 1,185 | 932 | 2,117 | 10 | 2 | 12 | 1 | ... | 1 |
| Hospitals . | 1,075 | 817 | 1,892 | 127 | 131 | 258 | 2,150 | 495 | 420 | 915 | 368 | 376 | 744 | 174 | 215 | 389 | 106 | 63 | 169 | 1 | ... | 1 | ... | ... | |
| Metropol. Licensed Houses . | 727 | 653 | 1,380 | 163 | 410 | 573 | 1,953 | 481 | 477 | 958 | 237 | 329 | 566 | 115 | 156 | 271 | 125 | 88 | 213 | ... | 2 | 2 | ... | ... | ... |
| Provincial Licensed Houses . | 921 | 717 | 1,638 | 284 | 228 | 512 | 2,150 | 398 | 416 | 814 | 296 | 258 | 554 | 116 | 142 | 258 | 91 | 58 | 149 | 3 | 1 | 4 | ... | ... | ... |
| Totals . | 2,831 | 2,291 | 5,122 | 8,843 | 10,889 | 19,723 | 24,845 | 4,460 | 4,495 | 8,955 | 2,266 | 2,686 | 4,952 | 1,363 | 1,775 | 3,138 | 1,507 | 1,141 | 2,648 | 14 | 5 | 19 | 1 | ... | 1 |

| | Patients Remaining 1st January, 1862. | | | | | | | | | | | | | | | | | | |
|---|---|---|---|---|---|---|---|---|---|---|---|---|---|---|---|---|---|---|---|
| | Private. | | | Pauper. | | | Total Lunatics. | Number deemed Curable. | | | Found Lunatic by Inquisition. | | | Criminals. | | | Chargeable to Counties or Boroughs. | | |
| | M. | F. | Total. | M. | F. | Total. | | M. | F. | Total | M. | F. | Total | M. | F. | Total | M. | F. | Total |
| County and Borough Asylums . . . | 155 | 112 | 267 | 8,758 | 10,630 | 19,388 | 19,655 | 941 | 1,272 | 2,213 | 8 | 5 | 13 | 272 | 111 | 383 | 878 | 1,012 | 1,890 |
| Hospitals . . . . . . . . . . . | 1,096 | 794 | 1,890 | 127 | 135 | 262 | 2,152 | 169 | 209 | 378 | 22 | 19 | 41 | 116 | 18 | 134 | ... | ... | ... |
| Metropolitan Licensed Houses . . . | 781 | 656 | 1,437 | 228 | 467 | 695 | 2,132 | 129 | 179 | 308 | 66 | 69 | 135 | 19 | 3 | 22 | 29 | 40 | 69 |
| Provincial Licensed Houses . . . . | 923 | 733 | 1,656 | 293 | 312 | 605 | 2,261 | 163 | 182 | 345 | 79 | 47 | 126 | 209 | 55 | 264 | 30 | 15 | 45 |
| Totals . . . . . . . . . . . | 2,955 | 2,295 | 5,250 | 9,406 | 11,544 | 20,950 | 26,200 | 1,402 | 1,842 | 3,244 | 175 | 140 | 315 | 616 | 187 | 803 | 937 | 1,067 | 2,004 |

Included in Total Lunatics.

The following are the original judgments, or orders, under which the lunatics were detained, with the proportion per cent. which the numbers under each bear to the whole:—

| | Number. | Proportion per cent. to whole Number. |
|---|---|---|
| Found insane | 131 | 13·5 |
| Acquitted insane | 140 | 14·4 |
| Insane, committed by justices | 88 | 9·1 |
| Dangerous lunatics, committed by justices | 7 | 0·7 |
| Convicts becoming insane after trial, and removed by order of Secretary of State | 604 | 62·3 |
| | 970 | 100·0 |

The following table shows the periods for which those detained for each crime have been under detention:—

| | Number of Persons. | Proportion per cent. of whole Number. |
|---|---|---|
| One year and under | 189 | 19·5 |
| Two years and above one | 199 | 20·5 |
| Three years and above two | 121 | 12·5 |
| Five years and above three | 128 | 13·2 |
| Ten years and above five | 203 | 20·9 |
| Fifteen years and above ten | 60 | 6·2 |
| Twenty years and above fifteen | 41 | 4·3 |
| Above twenty years | 29 | 2·9 |

The offences for which the lunatics were in custody in 1861 are shown in the accompanying table, the greatest numbers being,—for *larcenies*, 2341, or 24·1 per cent. of the whole; for *murder*, 141, or 14·5 per cent. of the whole; and for *attempts to murder*, 98, or 10·1 per cent. of the whole.

| | Male. | Female. | Total. |
|---|---|---|---|
| Murder | 96 | 45 | 141 |
| Attempts to murder, mania, stabbing. | 88 | 10 | 28 |
| Concealing birth, infanticide | 0 | 3 | 3 |
| Manslaughter | 4 | 3 | 7 |
| Rape | 7 | 0 | 7 |
| Assault with intent to ravish | 11 | 0 | 11 |
| Unnatural offences | 11 | 0 | 11 |
| Treasonable and seditious offences | 4 | 0 | 4 |
| Assaults | 42 | 4 | 46 |
| Indecent exposure of person | 6 | 0 | 6 |
| Burglary and housebreaking | 53 | 2 | 55 |
| Robbery on the highway, &c. | 12 | 0 | 12 |
| Sheep stealing | 7 | 0 | 7 |

| | Male. | Female. | Total. |
|---|---|---|---|
| Horse stealing | 7 | 0 | 7 |
| Larceny and petty thefts | 156 | 78 | 234 |
| Frauds and embezzlements | 5 | 0 | 5 |
| Receiving stolen goods | 5 | 1 | 6 |
| Arson and malicious burning | 36 | 5 | 41 |
| Wilful damage and other malicious offences | 9 | 7 | 16 |
| Forgery | 4 | 1 | 5 |
| Uttering counterfeit coin | 7 | 3 | 10 |
| Riot and breach of the peace | 35 | 9 | 44 |
| Under the vagrant laws | 42 | 12 | 54 |
| Dangerous persons at large | 5 | 0 | 5 |
| Deserters from the army or navy | 7 | 0 | 7 |
| For want of sureties | 39 | 16 | 55 |
| Other offences | 49 | 21 | 70 |

The total charges for criminal lunatics in the year were 26,701*l.* 6s. 11d., being an increase of 1,160*l.* 3s. 10d., or about 4·5 per cent. upon the total charges for the preceding year.

### *Lunacy Legislation*, 1862.

In the course of the past session of Parliament two important Acts were passed affecting lunatics: the "Lunacy Regulation Act," and the "Lunacy Acts Amendment Act." The following is a brief summary of the chief provisions of these Acts:—

*The Lunacy Regulation Act*, 1862 (25 and 26 Vict., cap. 86).—This Act is an amendment of the law contained in the Lunacy Regulation Act, 1853 (16 & 17 Vict. cap. 70), and is to be read and construed with the aid of the last-mentioned statute.

The inquiry to be made under every order for inquiry or commission of lunacy or issue, is to be confined to the question whether or not the person who is the subject of the inquiry is, *at the time of such inquiry*, of unsound mind and incapable of managing himself or his affairs, and no evidence as to anything done or said by such person, or as to his demeanour or state of mind, at any time being more than two years before the time of such inquiry, shall be receivable in proof of insanity on any such inquiry, or on the trial of any traverse of an inquisition, unless the judge or master shall otherwise direct. Under the Act of 1853 (sect. 4) the Lord Chancellor had power to direct an inquiry from what time the alleged lunatic had been of unsound mind. This power, therefore, is now abolished.

Wherever under the Act of 1853 the Lord Chancellor shall order an inquiry before a jury, he may henceforth direct an issue to be tried in one of the Courts of Common Law, and the question on such issue shall be whether the alleged insane person is of unsound mind and incapable of managing himself or his affairs.

On the trial of every such issue, the alleged lunatic shall, if within the jurisdiction, be examined before the taking of the evidence is commenced and at the close of the proceedings, before the jury consult as to their verdict, unless the judge shall otherwise direct, and such examinations shall take place either in open court or in private, as the judge shall direct.

No person is to be entitled to a traverse of any inquisition made upon the oath of a jury; but the Lord Chancellor, upon petition presented within three months after the trial of any such issue, may order a new trial, subject to such directions and upon such conditions as he may think proper. (scet. 7.)

The demand for an inquiry by jury may henceforth be made or withdrawn orally or by petition as well as by notice.

The Lord Chancellor may, upon consent of the lunatic and such other persons (if any) whose consent he may deem necessary, order the Commission to be superseded, on such terms and conditions as he may think proper.

It shall be lawful for the Lord Chancellor to order the costs of a petition for a Commission, and of the prosecution of any proceedings consequent upon such Commission, to be paid either by the petitioner or by the opponents, or out of the estate of the lunatic, and partly in one way and partly in another.

Where a lunatic does not oppose, and his property does not exceed 1000*l.* in value or 50*l.* per annum, the Lord Chancellor may apply it for his benefit in a summary manner without inquisition. And for the purpose of giving effect to any such order the Lord Chancellor may order land or other property of the lunatic to be sold or mortgaged for his benefit.

The last-mentioned power is also to be applicable to the property of persons acquitted on the ground of insanity.

The Lord Chancellor is also empowered to direct by order, any estate of a lunatic, whether in possession, reversion, remainder, contingency, or expectancy, to be charged for his maintenance, debts, or cost.

With respect to the visiting of lunatics, the visitors are to visit in such rotation and manner, and are to make such inquiries as the Lord Chancellor shall, by general order, direct, provided that after the 1st October, every lunatic shall be personally visited, and seen four times a year, and such visits are to be so regulated as that the interval between successive visits shall in no case exceed four months; but lunatics resident in licensed houses, asylums, or registered hospitals, shall not necessarily be visited by any of the visitors more than once a year.

The visitors are also to visit alleged lunatics, as the Lord

Chancellor may direct, and are to report to the Lord Chancellor once every six months.

The Lord Chancellor is empowered to allow pensions to the present medical visitors, not exceeding one half of their respective salaries. The visitors hereafter appointed, are to hold office during good behaviour, and are to receive salaries of 1,500*l.* a year, and shall not practise.

Superannuation allowances may be paid to any officer in lunacy who has served twenty years, and who shall be above sixty years of age.

*The Lunacy Acts Amendment Act*, 1862 (25 & 26 Vict. cap. 3.)—This Act is an amendment of the law relating to lunatics contained in the 8 & 9 Vict. cap. 100, 16 & 17 Vict. cap. 96, and 16 & 17 Vict. cap. 97, and is to be construed as one Act with them, and words defined by the said Acts are to have the same meaning in this Act.

*Establishment of County Asylums.*—Where plans, estimates, or contracts agreed to by a committee of visitors are disapproved of by one or more, but not all of the Courts of Quarter Sessions whose approbation is required, each Court disapproving, is to report thereon to the Secretary of State, who, after inquiry, shall, by writing, direct the plan, estimate, or contract, with or without alteration, or such other plan, &c., for the like purpose, as he may think fit, to be carried into execution, and the decision of the Secretary of State shall be final.

Plans of asylums for pauper lunatics submitted to the Commissioners in Lunacy are to be accompanied with estimates.

Arrangements may be made for the reception and care in a workhouse of a limited number of chronic lunatics, selected by the superintendent of the asylum of the district, and certified by him to be fit and proper to be so removed.

The committee of visitors of any asylum may provide accommodation for the burial of pauper lunatics dying in the asylum.

*Licensed Houses.*—Houses not previously licensed are to be inspected by the Commissioners before being licensed by the justices; and notice of proposed alteration of licensed houses is to be given to Commissioners before the consent of the visitors is given to such alteration.

The physician, surgeon, or apothecary, residing in, or visiting the house of a non-resident proprietor, shall be approved by the licensing Commissioners, or justices.

The proprietor or superintendent of a licensed house may, with the previous consent of two of the licensing Commissioners or justices, entertain as a boarder for a specified time, any person who may have been a patient in any asylum or licensed house, or under care as a single patient, within five years preceding.

No person shall be detained in any workhouse, being a lunatic or alleged lunatic, beyond the period of fourteen days, unless in the opinion, given in writing, of the medical officer of the union that such person is a proper person to be kept in a workhouse, nor unless the accommodation in the workhouse is sufficient.

Persons signing orders for admission must have seen the patient within one month, and the time and place when the patient was last seen is to be stated in the order.

The following persons are prohibited from signing any certificate or order, for the reception of any private patient into any licensed or other house.

First, any person receiving any per centage on, or otherwise interested in, the payments to be made by or on account of any patient received into a licensed or other house.

Second, any medical attendant, as defined by the Lunacy Act, 8 & 9 Vict. c. 100.

Wherever possible, the name and address of one or more of the relations of a pauper lunatic are to be inserted in the order for admission, and in the event of the patient's death, notice is to be sent to such relation, or one of such relations.

Where any medical certificate upon which a patient has been admitted is deemed by the Commissioners to be defective, and is not amended within fourteen days after notice, the Commissioners may order the patient's discharge.

The documents now required to be sent to the Commissioners, after two clear days and before seven clear days from the day of the admission of any patient (except the statement now required to be subjoined to the notice of admission), are to be sent within one clear day from the day of admission, and the excepted statement is to be sent as heretofore.

Every licensed house may be visited at any time, and if within the immediate jurisdiction of the Commissioners, shall be visited twice in every year by one or more of the Commissioners, in addition to the visits now required to be made by two at least.

Any one or more of the Commissioners may at any time visit every asylum and hospital for lunatics, and every gaol in which there may be, or may be alleged to be any lunatic.

Any two of the Commissioners may order removal of lunatics froma workhouse to an asylum.

Any two of the Commissioners may visit any pauper lunatic or alleged lunatic, and may call to their assistance a physician, surgeon, or apothecary; and upon his certificate, according to Schedule F, they may order such lunatic or alleged lunatic to be received into an asylum.

The superintendent of every asylum shall once in each half year transmit to the guardians of the union, a statement of the condition of every pauper lunatic chargeable to the union.

The proprietor of every licensed house within the jurisdiction of visitors appointed by justices, shall, within three days after a visit by the Visiting Commissioner or Commissioners, transmit a true copy of the entries made by them or him in the Visitors' Book, the Patients' Book, and the Medical Visitation Book, to the Clerk of the Visitors as well as to the Commissioners.

The Visiting Committee of every Union are to enter observations in a book to be provided, respecting dietary, accommodation, and treatment of lunatics in workhouses.

*Miscellaneous clauses.*—Patients may be permitted to be absent on trial from hospitals and private houses.

Two of the Commissioners, Governors, or Visitors, may of their own authority permit any pauper patient to be absent upon trial, for such period as they may think fit, and may make or order to be made an allowance to such pauper, not exceeding what would be the charge for him in the hospital, which allowance shall be charged for him as if he were actually in the hospital, but shall be paid over to him or for his benefit, as the Commissioners or visitors may direct.

In case any person allowed to be absent on trial do not return at the expiration thereof, and a medical certificate certifying that his detention is no longer necessary be not sent, he may at any time within fourteen days after the expiration of the same period be retaken, as in case of an escape.

Every letter written by a private patient in any asylum, hospital, or licensed house, or by any single patient, and addressed to the Commissioners, or Committee, or Visitors shall be forwarded unopened.

Every letter written by a private patient in any asylum, &c., or by a single patient, and addressed to any person other than the Commissioners, or Committee, or visitors, shall be forwarded, unless the superintendent or person having the charge of the patient prohibit the forwarding of such letter, by endorsement to that effect on the letter, in which case he shall lay all letters so endorsed before the Visiting Commissioners' Committee, or Visitors on their next visit.

Notice of the death of every patient to be sent to the Coroner.

Any two or more Commissioners or visitors may examine witnesses on oath.

*Pauper Lunacy, and Total Reported number of Lunatics in* 1860.—The 14th Annual Report of the Poor Law Board, just published, gives the following account of the amount of pauper lunacy in 646 unions and single parishes under Boards of Guardians, on the 1st January, 1861.

| | | | |
|---|---|---|---|
| Total number of insane paupers . . . . . | | | 32,920 |
| *Lunatics* :—Males . . . | 10,071 | | |
| Females . . . | 13,516 | | |
| | | | 23,587 |
| *Idiots* :—Males . . . . | 4443 | | |
| Females . . . | 4991 | | |
| | | | 9,333 |

These lunatics were provided for as follows :—
17,373 in County or Borough Lunatic Asylums.
889 in Registered Hospitals or Licensed Houses.
8543 in Union or Parish Workhouses.
758 in Lodgings or boarded out.
5357 Residing with Relatives.

The returns of the Commissioners in Lunacy for the 1st January, 1861, gave the number of pauper lunatics in asylums, hospitals, and licensed houses as 19,718. The whole number of existing unions do not, however, report to the Poor Law Board, and hence, in all probability, the difference in the two returns. Adopting the figures of the returns published by the Commissioners in Lunacy, the total number of pauper lunatics on the 1st January, 1861, would be 34,376. But even this number would not include the lunatics in several workhouses.

The number of lunatics who were private patients at the same date was 5116. Thus the total amount of reported lunatics at that period was 39,492—a number, from its including pauper lunatics in workhouses, boarded out, and receiving relief at home, 14,658 in excess of that reported by the Commissioners in Lunacy.

The returns of the Poor Law Board for the 1st January, 1859, show that at that time the number of pauper lunatics reported to them was 30,318, of whom 21,432 were lunatics, and 8886 idiots. Thus in 1860 there would appear to have been an increase of 2602 insane paupers, 2155 lunatics, and 447 idiots.

The total number of paupers receiving relief on the 1st January, 1861, was 891,868.

### SCOTLAND.

The fourth Annual Report of the Commissioners of Lunacy for Scotland shows the amount and distribution of known lunacy in that portion of the kingdom in 1860.

The total number of insane in Scotland, on the 1st January, 1861, including single patients, (approximatively estimated), was 8136, of whom 3914 were males and 4222 females; 2879 private, and 5257 pauper patients. On the 1st January, 1860, the corresponding numbers of private and pauper patients were 2858

and 5226. There was thus, in 1860, an increase of 21 of the former class of patients, and 31 of the latter. This increase in the number of pauper patients is unusually small. In 1858 the additional amount was 243; and in 1859, 246. The distribution of these patients was as follows:—

*Public and District Asylums.*

| | |
|---|---|
| Number of patients . . . . . . . | 2712 |
| Males . . . . . . . . . | 1353 |
| Females . . . . . . . . . | 1359 |
| Increase since 1st January, 1860 . . . . | 80 |
| Supported by private funds . . . . | 766 |
| „ by parochial rates . . . . | 1946 |

On the 1st January, 1860, the numbers supported by private funds and parochial rates were respectively 773 and 1859. It thus appears that during 1860 a decrease of 7 had taken place in the number of private patients, and an increase of 87 in that of paupers.

*Private Asylums.*

| | |
|---|---|
| Number of patients . . . . . . . | 907 |
| Males . . . . . . . . . | 378 |
| Females . . . . . . . . . | 529 |
| Increase since 1st January, 1860 . . . . | 55 |
| Supported by private funds . . . . . | 224 |
| „ by parochial rates . . . . . | 683 |

On the 1st January, 1860, the numbers supported by private funds and parochial rates were respectively 196 and 656. Thus during 1860 an increase of 28 had taken place in the number of private patients, and one of 27 in that of paupers.

*Poor-houses.*

| | |
|---|---|
| Number of patients . . . . . . . | 843 |
| Males . . . . . . . . . | 343 |
| Females . . . . . . . . . | 500 |
| Decrease since 1st January, 1860 . . . . | 23 |

With the exception of the females, the whole of these patients were maintained by parishes.

*Single Patients.*

| | |
|---|---|
| Number . . . . . . . . . | 1787 |
| Males . . . . . . . . . | 799 |
| Females . . . . . . . . . | 988 |
| Decrease since 1st January, 1860 . . . . | 60 |
| Living with relatives:— | |
| Males . . . . . . . | 645 |
| Females . . . . . . . | 739 |
| Living with strangers:— | |
| Males . . . . . . . | 136 |
| Females . . . . . . . | 192 |
| Living alone:— | |
| Males . . . . . . . | 18 |
| Females . . . . . . . | 57 |

"During our first inspection of the insane left in their houses," the Commissioners state, "we acquired knowledge of the existence of 1887 private single patients—1041 males and 946 females; but in later inspections we have forborne to make any searching inquiries respecting such patients, as, for the most part, they are living with their families, and, consequently, are not subject to statutory visitation. As it is probable, however, that the decrease in their numbers by removal to asylums, recovery, and death is at least compensated by the occurrence of new cases, and by the transference home of unrecovered patients from asylums, we continue to adopt the numbers formerly ascertained as still sufficiently accurate to be adopted in a general estimate of the number of the insane in Scotland."

The following table shows the admissions into public and private asylums, and the lunatic wards of poor-houses, the average number of patients resident, the discharges and deaths in 1860.

| Asylums. | Admitted. | | Average numbers resident. | | Discharged. Recovered. | | Discharged. Not recovered. | | Dead. | |
|---|---|---|---|---|---|---|---|---|---|---|
| | M. | F. | M. | F. | M. | F. | M. | F. | M. | F. |
| Public . . . . . . | 442 | 518 | 1351·0 | 1319·0 | 167 | 208 | 124 | 130 | 139 | 99 |
| Private . . . . . | 128 | 177 | 363·0 | 515·5 | 43 | 66 | 36 | 42 | 20 | 42 |
| Poor Houses . . . . | 163 | 174 | 336·5 | 498·5 | 72 | 94 | 31 | 43 | 63 | 49 |
| Total . . . . . | 735 | 869 | 2050·5 | 2332·0 | 282 | 368 | 191 | 215 | 222 | 190 |

Much statistical data of considerable interest is contained in the Report, although not of a nature admitting of ready condensation. The figures given would seem to indicate that there is a progressive decline in the rate of increase of pauper lunacy, and a stationary condition of private lunacy; that the increase of known insanity is most probably due to the accumulation of the insane, and not in any marked degree to a greater disposition in modern times to mental disease; and that lunacy is of more frequent occurrence in urban than in rural districts. The Commissioners add, however, that "it is quite possible the appearance of a greater occurrence of insanity in towns than in the country may be altogether delusive, and may be entirely dependent on repeated intimations given of the same cases, and on the different interpretation given to the term lunacy in different districts or parishes." "The constant growth of pauper lunacy, and stationary condition of private lunacy," the Commissioners also say, "deserve most serious attention, and, as was pointed out in our last Report, indicate either that a larger number of private patients are improperly removed from asylums, or that a large number of pauper patients are unnecessarily detained in such establishments."

The number of *criminal lunatics* returned on the 1st of January of the present year was 29—18 males and 11 females. They are confined in the general prison at Perth.

The Commissioners express their deep regret at the loss by death of one of the Deputy Commissioners, Dr. Cockburn.

---

